# Metastatic Cervical Cancer to the Duodenum: A Learning Point

**DOI:** 10.7759/cureus.13874

**Published:** 2021-03-14

**Authors:** Javier Ash, Alice Tsai

**Affiliations:** 1 General Surgery, Princess Alexandra Hospital, Harlow, GBR; 2 General Surgery, Croydon University Hospital, London, GBR

**Keywords:** uterine cervical cancer, duodenum, rare metastases

## Abstract

An elderly woman was admitted to the hospital with generalised abdominal pain and bowel obstruction symptoms in a background of renal cell carcinoma and cervical cancer. Investigations showed a degree of gastric outlet obstruction with mild distension of the small bowel loops with no lead point seen and a raised alkaline phosphatase (ALP). Oesophago-gastroduodenoscopy (OGD) showed a stricture at D1/D2; therefore, an enteric stent was inserted. Biopsies showed metastatic cervical cancer. A few cases of metastatic cervical cancer to the duodenum have been reported. Obstructive bowel symptoms in the background of cervical cancer should raise the possibility of metastases in future practice.

## Introduction

Carcinoma of the cervix is the second most common gynaecological malignancy [1], and in the United Kingdom (UK), it is the 14th most common cancer accounting for 2% of all new cancer cases in females [2].

Carcinoma of the cervix usually spreads in a predictable manner with most via a direct extension to surrounding structures such as the vagina, peritoneum and bladder [1]. Distant metastasis occurs secondary to lymphatic and haematogenous spread to the liver, lung and bone marrow [1,3].

Metastatic spread to the duodenum from cervical cancer is rare, with only 15 reported cases in the existing literature identifying metastatic spread to the bowel [1,3-12].

Knowledge of rarer presentations of common pathologies is crucial to mitigate diagnostic overshadowing and ensure the breadth of differential diagnoses. As treatment for malignancies improve and patients live longer with known malignancies, rarer presentations and metastatic sites may be more frequently reported. These may guide research into the spread of cancer and thus how better to treat it in the future.

## Case presentation

An 81-year-old Caucasian woman presented with a five-day history of severe generalised abdominal pain, which was colicky in nature with non-bile-stained vomiting, distention, and two days of not having opened her bowels. The abdomen was soft, with general tenderness and guarding in the right upper quadrant. There was no relevant family and social history.

She was diagnosed three years previously with squamous cell carcinoma of the cervix with bladder involvement (Stage IVa on the International Federation of Gynaecology and Obstetrics staging - FIGO), in which she underwent external radiotherapy and intrauterine balloon therapy. She also had a common bile duct stent inserted for obstruction secondary to a pancreatic lesion in the uncinate process of unknown aetiology.

Past medical history further included a left nephrectomy for renal cell carcinoma five years previously, a ureteric stent after an episode of hydronephrosis secondary to a renal stone, chronic kidney disease and hypertension.

The patient was alert although in some discomfort. Respiratory examination elicited good air entry bilaterally with no added sounds. Cardiovascular examination sounds S1 and S2 present with no additional sounds. The abdomen was soft but distended with generalised tenderness and guarding in the right upper quadrant. No masses or herniae were elicited and there was no sign of peritonism or rebound tenderness. There was no evidence of pitting oedema, and calves were soft. Digital rectal examination showed dark stool but no blood visible, with no palpable masses. Laboratory-based investigations showed a raised alkaline phosphatase (1035IU/L) but were otherwise within normal limits.

**Figure 1:**
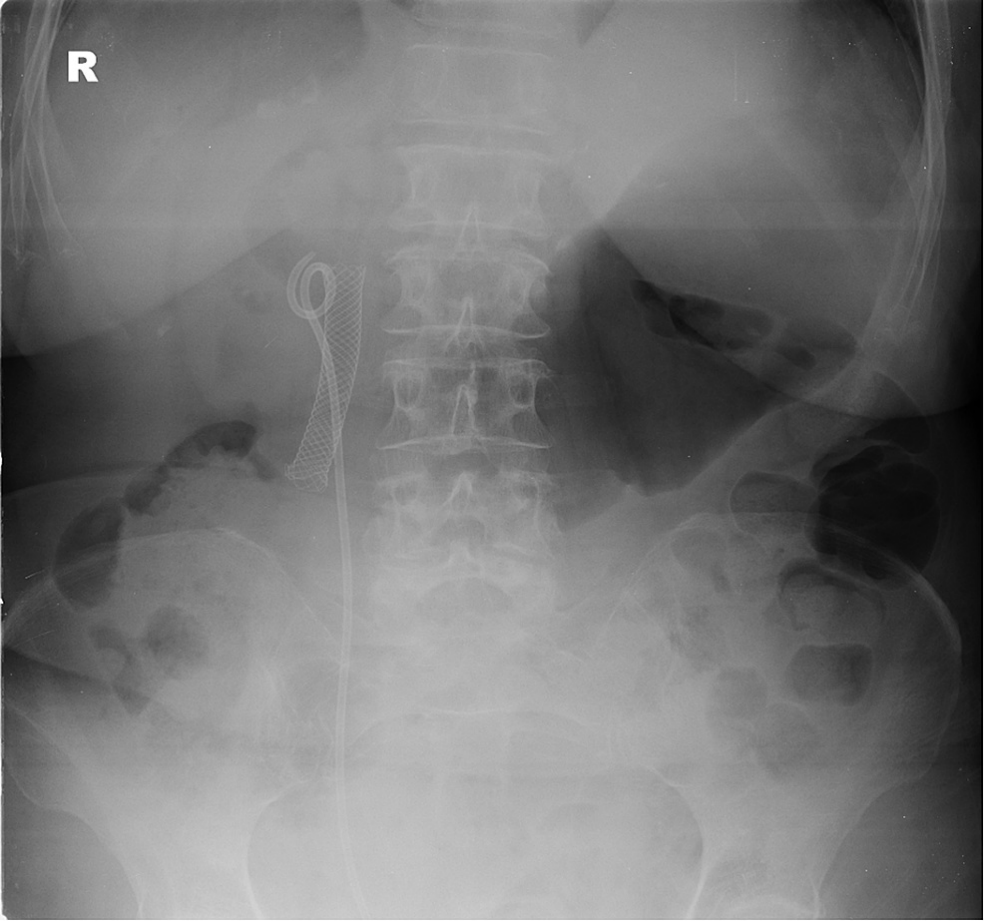
Abdominal radiograph on admission

The abdominal radiograph (Figure 1) showed that the stomach was markedly distended and the fundus was fluid-filled. The small bowel pattern was normal. Acute colonic abnormality was absent. There was a metal biliary stent in situ with the presence of gas in the biliary tree. Appearances raised the possibility of gastric outlet obstruction.

**Figure 2:**
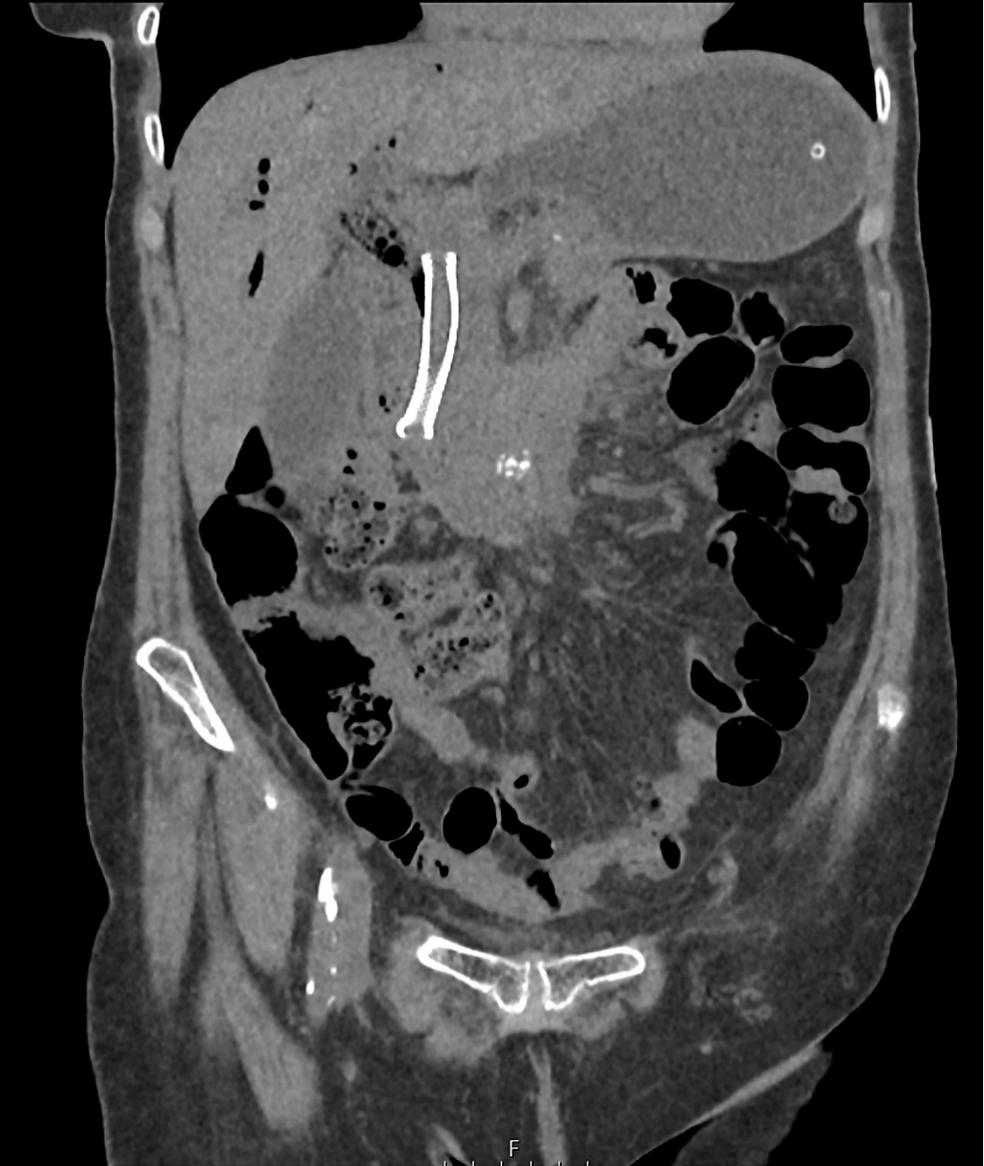
Computed tomography of the patient on admission: coronal plane

Computed tomography (CT) of the abdomen and pelvis (Figure 2) showed the following: “There is increased soft tissue seen around the bile duct stent. Non-specific mild distension of small bowel loops proximally with no lead point seen.”

The patient was diagnosed with gastric outflow obstruction of an unknown cause. It was suggested that this could be due to a known pancreatic lesion of unknown aetiology or new malignancy. The CT scan and normal bilirubin level also confirmed the biliary system's patency; the raised alkaline phosphatase (ALP) may indicate liver or bone metastases.

A Ryles tube was inserted for decompression, and the patient was discussed at the radiology and upper gastrointestinal multi-disciplinary team meeting. The patient underwent oesophago-gastroduodenoscopy (OGD), which showed a stricture at D1-D2. Biopsies of the lesion were taken and a 12-cm stent was inserted with the most proximal aspect at the duodenal bulb.

Biopsies showed lymphovascular permeation of the mucosa and submucosa by nests of tumour cells resembling squamous cells. The cells were P16 and P63 positive, in keeping with squamous cell carcinoma. A fluorescence in situ hybridization (FISH) analysis of the sample detected human papillomavirus 16.

Post-enteric stent insertion, the oral intake was slowly re-introduced after a confirmatory radiograph of the stent position. The patient had episodes of vomiting and a barium meal was performed, which showed the stent in situ with contrast passing freely. It was noted that the stent lumen was severely narrowed at the level of the second and third part of the duodenum; however, the bolus could pass well.

Post-procedure, there was a noted increase in the patients' ALP (3245IU/L). Ultrasound of the abdomen showed a dilated intrahepatic and common bile duct. The patient was offered a percutaneous transhepatic cholangiogram to relieve the blockage for symptom relief, however, the patient refused further interventions. It is believed that the enteric stent may have interfered with the patency of the previously placed biliary stent. The patient was discharged home to continue with palliative care in the community. The patient subsequently passed away at home due to her illness.

## Discussion

Of the 15 cases reported identifying metastatic spread to the bowel [1,3-12], three presented with upper gastrointestinal bleeding [1,3], one with small bowel perforation [3], one with a psoas abscess [12], one incidentally [11], and the rest with obstructive symptoms or pain [3-10]. The most common site within the small bowel tends to be the duodenum [1,3-9,11] with three cases in the ileum [1] and two in the jejunum [1,10]. Indirect small bowel obstruction via para-aortic lymph nodes caused by cervical metastases has also been reported [3,12].

The pathophysiology for this manner of transmission is poorly understood in the literature. Cervical cancer usually spreads via direct extension or via the lymphatics [1,3]. Primary cancer affecting the small bowel is rare with most cancer found predominantly secondary to metastasis [5]. Spread is usually via the para-aortic mesenteric lymph nodes, which do not explain the manner of spread from cervical cancer [5]. The literature review indicates that metastases seem to have a predilection for the duodenum, yet is uncertain as to the cause. Duodenal metastases from cervical carcinoma are rare, but recognising such abnormal presentations or pathological varieties is essential for ongoing best medical practice. The pathology can present in several ways; however, it should be noted that epigastric pain and obstructive symptoms on a cervical cancer background should bring suspicion for metastases.

Further research in this field could investigate the manner of transmission of cervical cancer to the bowel, possibly questioning why such a predilection for the duodenum appears to occur. Furthermore, more research needs to be undertaken to better understand the most effective management of this condition.

## Conclusions

Although rare, cervical cancer can spread to the bowel with a predilection for the duodenum. Duodenal metastasis via cervical cancer most commonly presents with epigastric pain and obstructive symptoms, and so clinicians should be alert to patients' past medical history to ensure diagnostic breadth in differential diagnoses. Duodenal stents are appropriate in the alleviation of symptoms for the presentation.
